# Epidermoid cyst in an intrapancreatic accessory spleen diagnosed by contrast-enhanced EUS and EUS-FNA (with video)

**DOI:** 10.1097/eus.0000000000000047

**Published:** 2024-03-07

**Authors:** Ryogo Minami, Jun Nakahodo, Hiroki Tabata, Kazuro Chiba, Shinichiro Horiguchi, Terumi Kamisawa

**Affiliations:** 1 Department of Gastroenterology, Tokyo Metropolitan Cancer and Infectious Diseases Center, Komagome Hospital, Tokyo, Japan; 2 Department of Pathology, Tokyo Metropolitan Cancer and Infectious Diseases Center, Komagome Hospital, Tokyo, Japan.

Epidermoid cyst in an intrapancreatic accessory spleen (ECIPAS) is a rare, pancreatic cystic lesion covered with epithelioid cells and surrounded by splenic tissue.^[1]^ Most cases of ECIPAS are diagnosed postoperatively based on pathological findings.^[2]^ There are few reports of ECIPAS diagnosed by contrast-enhanced EUS (CE-EUS) or EUS-FNA. We present herein a case of ECIPAS diagnosed by CE-EUS and EUS-FNA.

A 28-year-old, female patient was admitted with pancreatic cysts. Contrast-enhanced computed tomography revealed a 17-mm, multilocular cystic lesion and a 7-mm, unilocular cystic lesion in the pancreatic tail. The margin of the cysts had the same enhancement as the spleen in the arterial phase [Figure 1]. Magnetic resonance imaging (MRI) demonstrated slight hypointensity on T1-weighted imaging and slight hyperintensity on T2-weighted imaging. The peripheral portion of the cysts had no areas of signal hypointensity like that of the spleen on superparamagnetic iron oxide imaging (SPIO) [Figure 2]. EUS demonstrated well-demarcated, round, hypoechoic cystic lesions [Figure 3], which gradually enlarged. EUS 3 years later demonstrated no obvious, solid portion around the cysts [Figure 4], but CE-EUS demonstrated an enhanced, solid portion around the cysts, which was identical to the spleen [Figure 5] [Video 1] and was thought to be an intrapancreatic accessory spleen (IPAS). EUS-FNA of the solid portion was performed using a 22-G needle (EZ-shot 3; Olympus Medical Systems Corp, Tokyo, Japan). Histopathology revealed CD8-positive splenic sinus lining cells specific to the red splenic marrow [Figure 6]. Based on these findings, the solid portion and the cysts were diagnosed as IPAS and ECIPAS, respectively. The patient was monitored without surgical resection.

**Figure 1:**
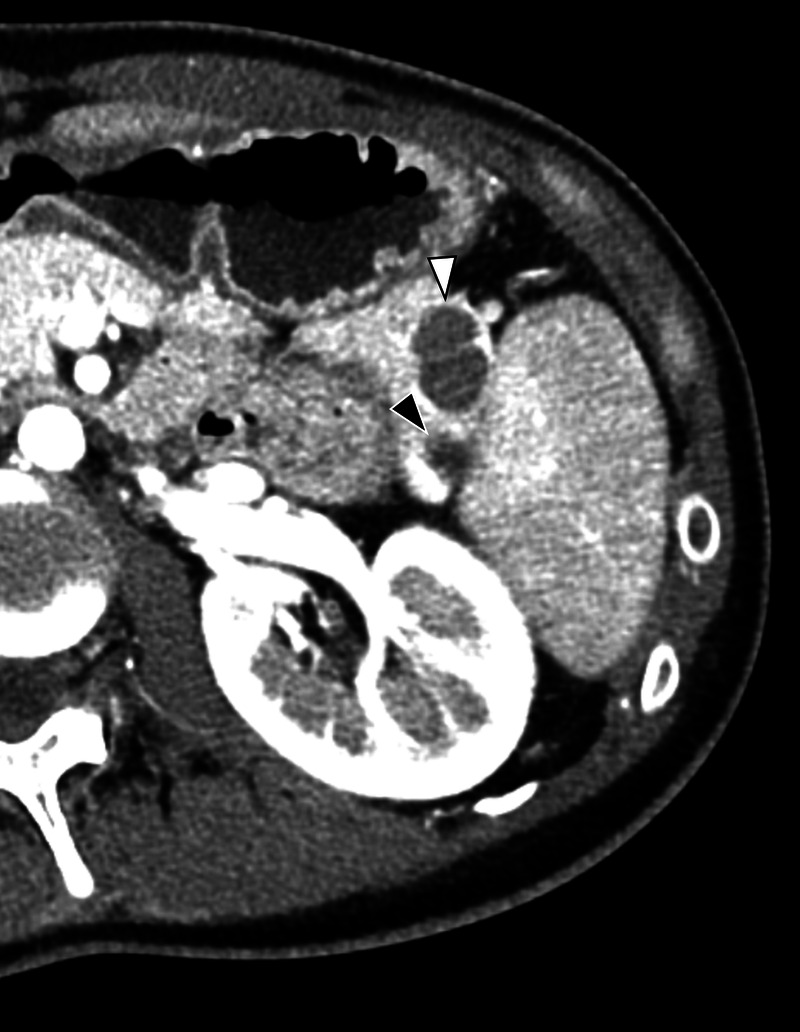
Contrast-enhanced computed tomography revealed a 17-mm multilocular cystic lesion (white arrow) and a 7-mm unilocular cystic lesion (black arrow) in the pancreatic tail. The margin of the cysts had the same enhancement as the spleen in the early arterial phase.

**Figure 2:**
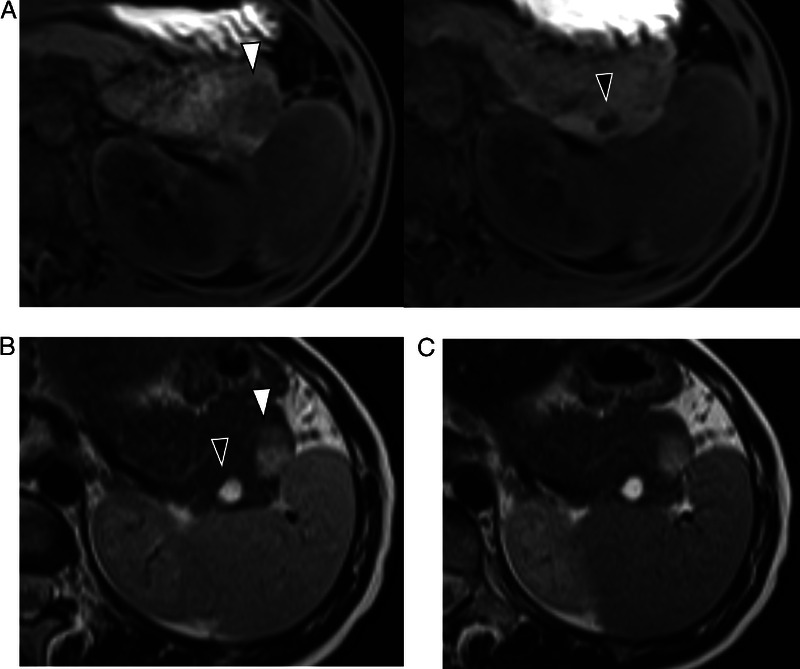
Magnetic resonance imaging. A, A T1-weighted imaging revealed areas of slight hypointensity. B, A T2-weighted imaging revealed areas of slight hyperintensity. C, The peripheral portion of the cysts had no areas of hypointensity like that of the spleen on superparamagnetic iron oxide imaging.

**Figure 3:**
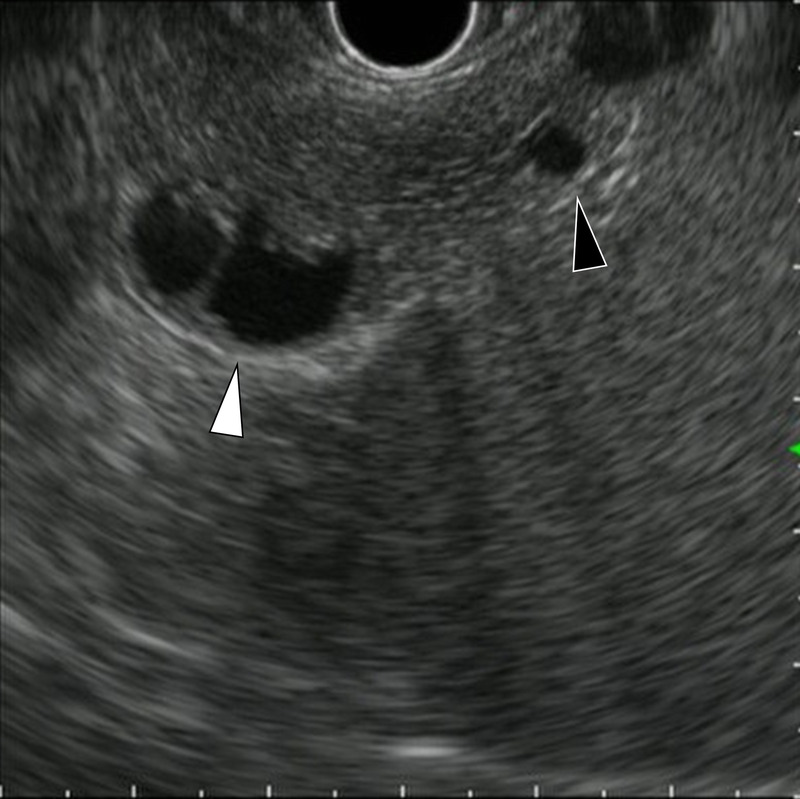
EUS demonstrated well-demarcated, round, hypoechoic cystic lesions in the pancreatic tail.

**Figure 4:**
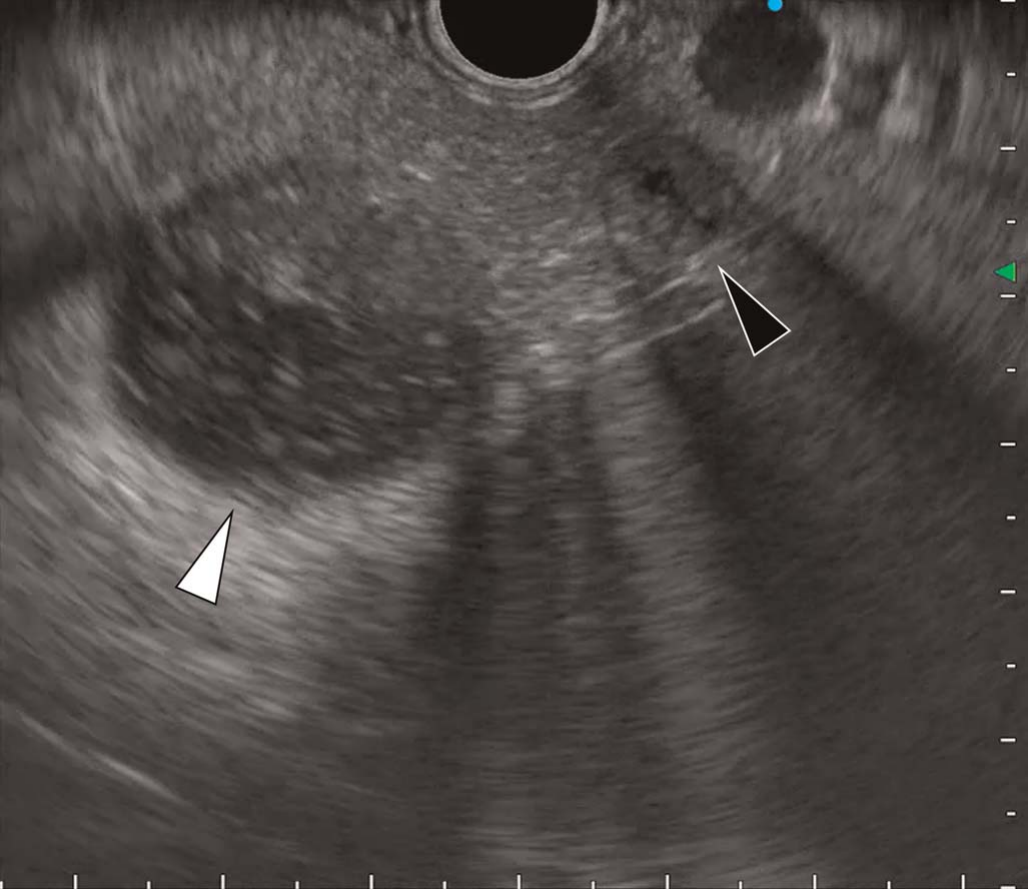
EUS 3 years later demonstrated no obvious solid portion around the cysts.

**Figure 5:**
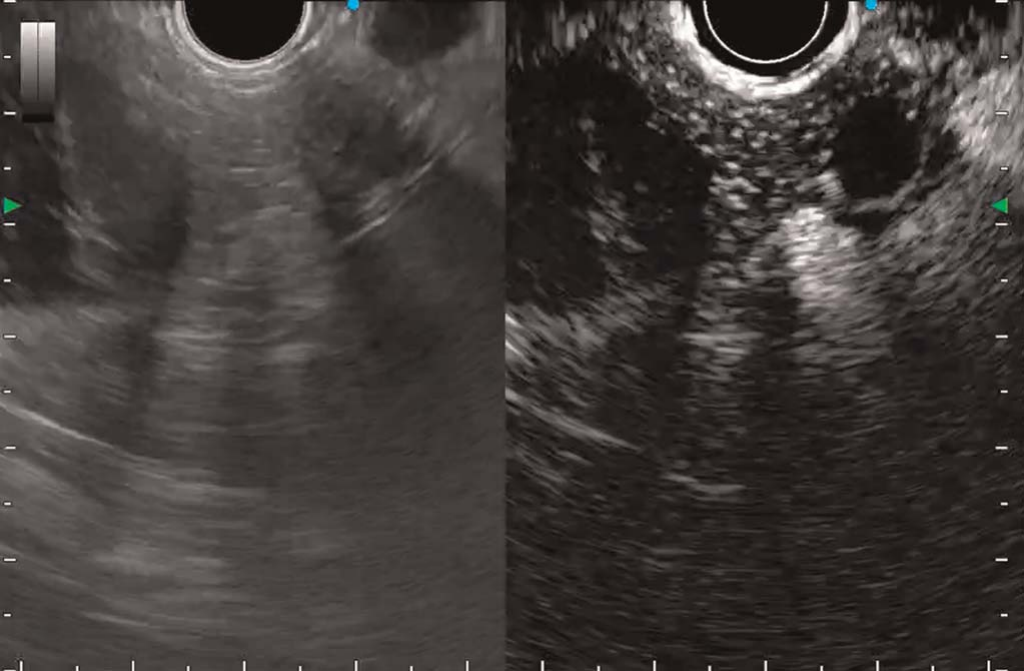
Contrast-enhanced EUS revealed an enhanced, solid portion surrounding the cysts.

**Figure 6:**
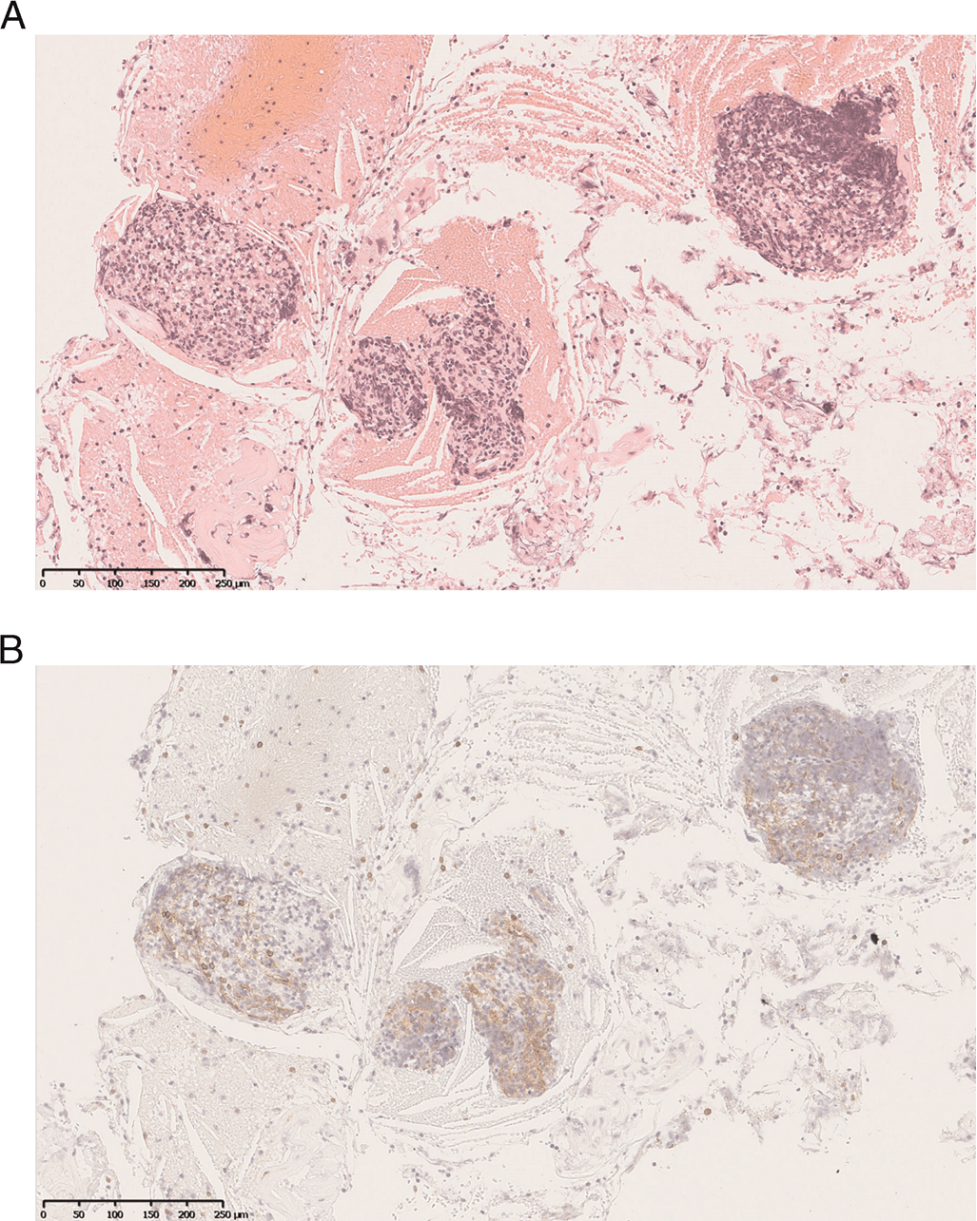
Histopathology of the EUS-FNA samples. A, Microvessel clusters were accompanied by numerous lymphocytes. Cholesterosis was visible in the surrounding area. B, Endothelial cells, considered to be splenic sinus lining cells, were positive for CD8 immunostaining. A, Hematoxylin and eosin staining; B, CD-8 immunostaining.

IPAS surrounding the cyst is key to accurate preoperative diagnosis of ECIPAS. The signal intensity of IPAS on MRI is similar to that of the spleen, with the SPIO image being especially useful for diagnosis ^[3]^. However, a radiographic diagnosis becomes difficult when the IPAS is compressed by cysts ^[4]^. The present case lacked MRI findings typical of IPAS, but CE-EUS was able to detect the lesion.

In conclusion, CE-EUS and EUS-FNA may be useful for diagnosing ECIPAS and avoiding surgical resection.

## Video Legend

Contrast-enhanced endoscopic ultrasonography (CE-EUS) demonstrated an enhanced, solid portion around the cysts, which was identical to the spleen.
